# Ultra-Early (<5 Hours) Decompression for Thoracolumbar Spinal Cord Injury: A Case Series

**DOI:** 10.7759/cureus.53971

**Published:** 2024-02-10

**Authors:** Matthew T Carr, Abhiraj D Bhimani, Jacques Lara-Reyna, Zachary L Hickman, Konstantinos Margetis

**Affiliations:** 1 Neurosurgery, Icahn School of Medicine at Mount Sinai, New York, USA

**Keywords:** ultra-early decompression, case series, neurological outcomes, early surgery, early decompression, spinal cord injury

## Abstract

Early surgical decompression within 24 hours for traumatic spinal cord injury (SCI) is associated with improved neurological recovery. However, the ideal timing of decompression is still up for debate. The objective of this study was to utilize our retrospective single-institution series of ultra-early (<5 hours) decompression to determine if ultra-early decompression led to improved neurological outcomes and was a feasible target over previously defined early decompression targets.

Retrospective data on patients with SCI who underwent ultra-early (<5 hours) decompression at a level one metropolitan trauma center were extracted and collected from 2015-2018. American Spinal Injury Association (ASIA) Impairment Scale (AIS) grade improvement was the primary outcome, with ASIA Motor score improvement and complication rate as secondary outcomes.

Four individuals met the criteria for inclusion in this case series. All four suffered thoracolumbar SCI. All patients improved neurologically by AIS grade, and there were no complications directly related to ultra-early surgery. Given the small sample size, there was no statistically significant difference in outcomes compared to a control group who underwent early (5-24 hour) decompression in the same period.

Ultra-early decompression is a feasible and safe target for thoracolumbar SCI and may lead to improved neurological outcomes without increased risk of complications. This case series can help create the foundation for future, larger studies that may definitively show the benefit of ultra-early decompression.

## Introduction

Traumatic spinal cord injury (SCI) is a major cause of morbidity worldwide, with estimates of age-adjusted global incidence of 130 per million persons [1]. The United States has a relatively high annual incidence of SCI compared to other countries, with an estimated 40.1 per million persons [2]. Total healthcare costs related to SCI care are substantial, with an estimated $7.7 billion annually in the United States alone [3]. Given the global and personal health burden of traumatic SCI and its sequelae, it remains an active area of research to improve neurological outcomes.

One promising treatment that has been linked to improved neurological recovery is early decompression following injury [4-6]. The STASCIS trial showed benefit in improvement of AIS grades for decompression <24 hours after injury in cervical SCI, and began a paradigm shift in the timing of decompression in SCI [5]. Thoracolumbar SCI is less well-studied, but also has substantial data in favor of early decompression. More recently, there is evidence that an earlier target for decompressive surgery can see greater improvement in neurological outcomes, particularly with urgent <8-hour decompression [7]. Another prior study has evaluated <5-hour decompression as an ultra-early surgical target in cervical SCI [8]. Timing of surgical decompression remains debated, however [7-9]. In SCI, there is ample literature supporting “time is spine”, and thus it deserves to be determined if there is an optimal earlier target for surgical decompression following SCI. We sought to determine whether a goal of ultra-early decompression (<5 hours) for SCI led to improved neurological outcomes. Secondarily, we evaluated the rate of complications in ultra-early surgery.

## Case presentation

Methods

Retrospective data for this case series were collected from medical records and the trauma database from New York City Health and Hospitals / Elmhurst, a level 1 trauma center in Queens, New York. The enrollment period was between May 2015 and April 2018. Inclusion criteria were SCI of grade A-D on the American Spinal Injury Association (ASIA) Impairment Scale (AIS), decompression within 5 hours of injury, preceding trauma, and age ≥18 years old.

Time of injury was obtained from emergency medical services (EMS) run reports. Time of decompression was not directly available in this retrospective study, and instead, we used time of surgical incision as an estimate. Decompression time was thus defined as the duration between time of injury to time of surgical incision. We classified ultra-early decompression as occurring <5 hours from injury.

Demographic variables collected included age, sex, race, and ethnicity. Pre-hospital data collected included injury date/time, injury mechanism, EMS dispatch date/time, EMS time of arrival at the scene, EMS time of leaving the scene, EMS time of arrival to the emergency department (ED), and vitals and Glasgow Coma Scale (GCS) in the field. Pre-operative hospital variables included diagnoses, injury level, AIS grade, ASIA motor scores, AOSpine classification, subaxial injury classification and severity (SLICS)/ thoracolumbar injury classification and severity (TLICS) scores, MRI brain and spinal injury center (BASIC) grades, levels of T2 edema, medical comorbidities, ethanol level, drug screen, initial ED vitals, initial ED GCS, injury severity score (ISS), and admitting service. Operative data collected included the operating attending surgeon, date/time of arrival to OR, time of incision, time operation ended, surgery performed, and intraoperative complications. SCI data collected included mean arterial pressure (MAP) goals, steroid use, steroid duration, steroid dosage, venous thromboembolism prophylaxis timing, postoperative complications, intensive care unit (ICU) length of stay (LOS), total hospital LOS, discharge date/time, discharge destination, discharge AIS and ASIA motor scores, 3-month AIS and ASIA motor scores, 6-month AIS and ASIA motor scores, and most recent AIS and ASIA motor scores.

T-tests were performed using SAS University Edition (Cary, NC, USA) with inferences made at the 5% level. The institutional review board reviewed this study and determined it to be exempt human research, and all research was conducted in accordance with the Helsinki Declaration. This case series has been reported in line with the PROCESS guidelines [10].

Results

**Figure 1:**
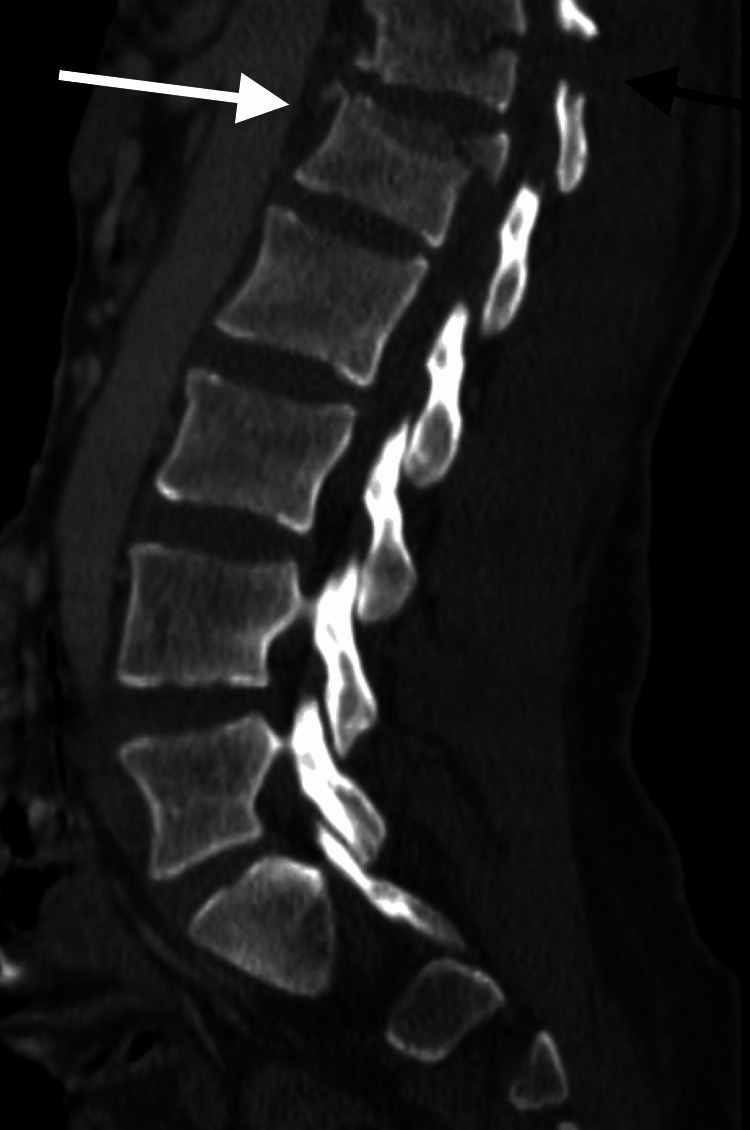
Preoperative sagittal CT for patient 1 demonstrates a bony Chance fracture (AOSpine subtype B1) through T12 (black arrow) and an incomplete burst fracture (AOSpine subtype A3) through the superior portion of L1 (white arrow).

**Figure 2:**
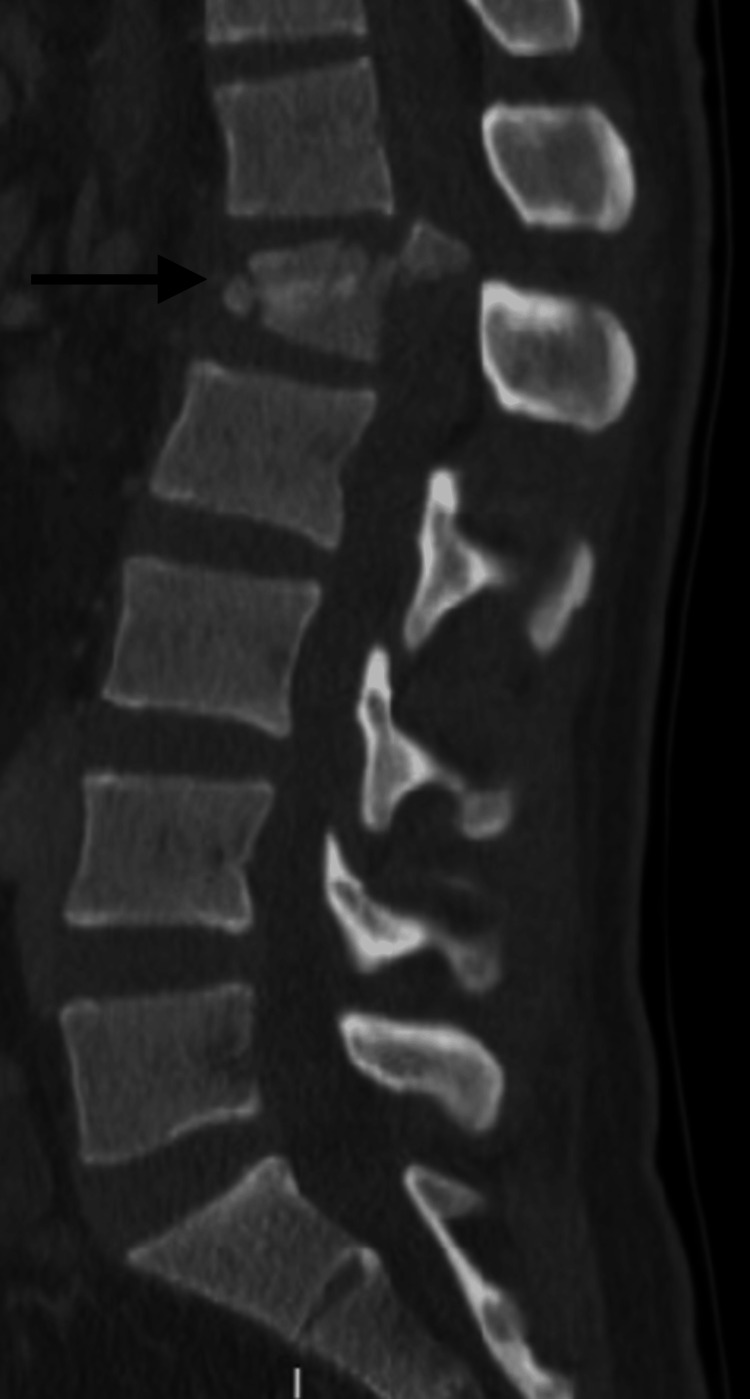
Preoperative sagittal CT of patient 2 shows a complete burst fracture (AOSpine subtype A4) of L1 (black arrow) with significant retropulsion.

**Figure 3:**
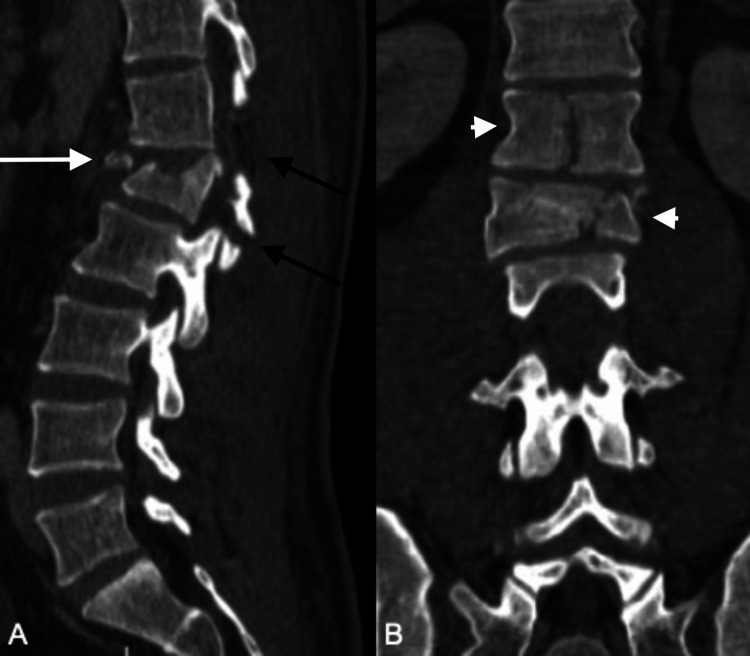
Preoperative sagittal (A) and coronal (B) CT of patient 3 highlights the osseoligamentous posterior tension band injury (AOSpine subtype B2) (black arrows) and significant complete burst fracture (AOSpine subtype A4) of L1 (white arrow). The complete burst fractures (AOSpine subtype A4) of T12 and L1 (white arrowheads) are also demonstrated on the coronal (B).

**Figure 4:**
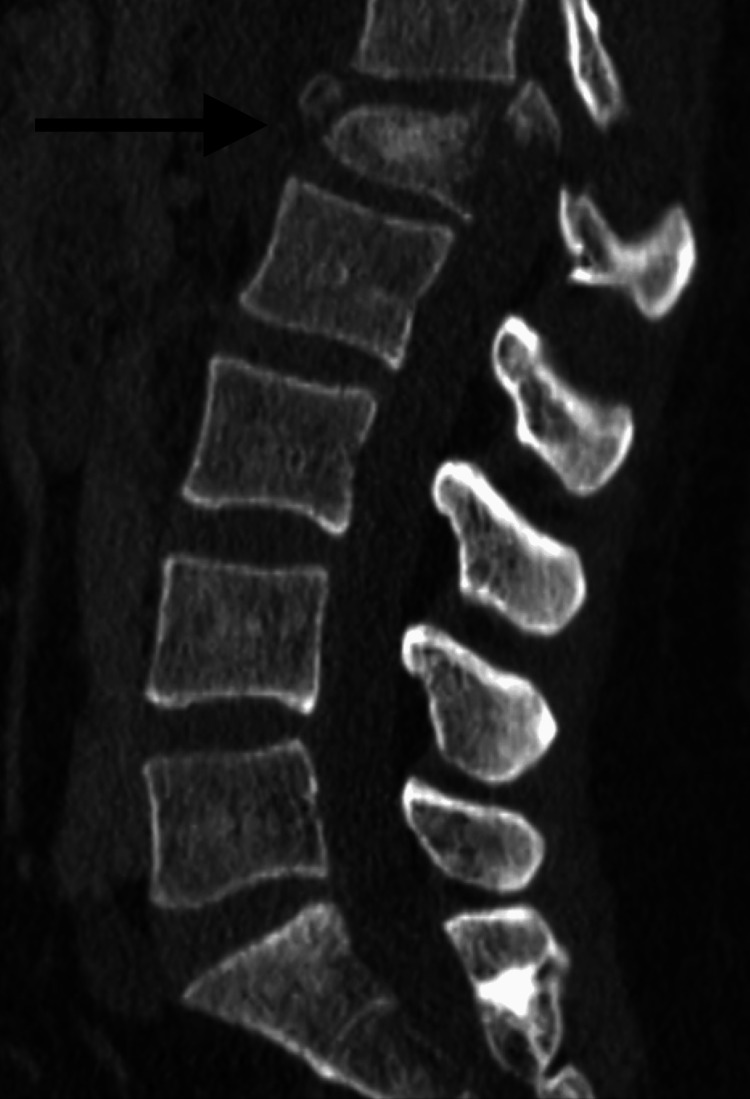
Preoperative sagittal CT of patient 4 demonstrates the complete burst fracture (AOSpine subtype A4) of L1 (black arrow) with significant bony retropulsion into the canal.

A total of four individuals met the criteria for inclusion. Demographics and sample characteristics are presented in Table 1. Mean age was 31.8 ± 8.4 years. The sample consisted of three males (75%). All individuals had thoracolumbar SCI. Median time to decompression was 4.1 hours, with a range from 3 hours 32 minutes to 4 hours 46 minutes. Initial AIS grade was B in three individuals (75%) and D in one individual (25%). All injuries were blunt injuries, all due to falls. ISS ranged from 17 to 25. Figures 1-4 show the preoperative CT scans for each patient in the series.

**Table 1:** Demographics for case series

Characteristics	N (%)
Age (range)	31.8 (20-38)
Male sex	3 (75%)
Race/Ethnicity	
Black	1 (25%)
White	1 (25%)
Asian	1 (25%)
Hispanic	1 (25%)
Admission AIS Grade	
B	3 (75%)
D	1 (25%)

The AIS is an ordinal scale, but it has been treated as a numerical scale in the literature, allowing for a more concise presentation of the results [11]. By doing so here, the mean AIS improvement in this series was 1.25 ± 0.50. We compared this to a sample of 11 patients over the same period who underwent early decompression (5-24 hours) where the mean AIS improvement was 0.82 ± 0.88, but this difference did not reach significance (p=0.38). Mean ASIA Motor improvement in this series was 25.75 ± 15.04, also not significantly better compared to our single institution early decompression group ASIA Motor improvement of 19.45 ± 28.58 (p=0.69).

Operative and medical treatment parameters for each person are noted in Table 2. No intraoperative or postoperative complications were directly related to ultra-early surgery. The overall hospital complications included one individual who had a superficial surgical site infection managed medically. Two individuals suffered a urinary tract infection. There were no mortalities in this case series.

**Table 2:** Neurological and patient characteristics for case series ASIA = American Spinal Injury Association. AIS = ASIA Impairment Scale. ISS = Injury Severity Score. MRI = Magnetic Resonance Imaging. MAP = Mean Arterial Pressure.

Patient Number	Age	Race	Ethnicity	Sex	Region of Injury	Level of Injury	Trauma to Decompression (mins)	Decompression Time (Hrs)	Surgical Procedure	Intraoperative Complications	Postoperative Complications	Admission AIS	Admission ASIA Motor	ISS	AOSpine Classification	Length of Follow-Up (months)	Post-Op AIS at Last Follow-Up	AIS improvement at Last Follow-Up	Post-Op ASIA Motor at Last Follow-Up	ASIA Motor Improvement	MRI Y/N	Steroids (Y/N)	MAP >85 duration (days)	Injury Mechanism
1	38	White	Non-Hispanic	Female	Thoracolumbar	T12	250	4.2	T11-12 laminectomies, T9-L3 posterior instrumented fusion	--	--	B	50	24	T12: B1 (L1: A3; N3)	6	C	1	69	19	N	Y	7 days	Fall
2	20	Asian	Non-Hispanic	Male	Thoracolumbar	L1	243	4.1	L1-2 laminectomies, T11-L3 posterior instrumented fusion	--	Urinary tract infection, Superficial surgical site infection	B	50	17	L1: A4 (N3)	16	D	2	91	41	N	Y	4 days	Fall
3	29	Other	Hispanic	Male	Thoracolumbar	T12-L1	286	4.8	T11-L1 laminectomies, T10-L3 posterior instrumented fusion	--	--	D	65	22	T12-L1: B2 (T12: A4; L1: A4; N3)	28	E	1	100	35	N	Y	4 days	Fall
4	38	Black	Non-Hispanic	Male	Thoracolumbar	L1	212	3.5	T12-L1 laminectomies, L1 transpedicular decompression, open fracture reduction, T11-L3 posterior instrumented fusion	--	Urinary tract infection	B	55	25	L1: A4 (N3)	1	C	1	63	8	N	Y	7 days	Fall

## Discussion

Ultra-early (<5 hours) surgical decompression for persons with traumatic SCI may be a feasible time frame to attain in thoracolumbar SCI. All patients undergoing ultra-early decompression experienced AIS grade and ASIA motor score improvement, but there was no significant difference compared to those who underwent the previously studied target of 24-hour early decompression. The ultra-early surgery individuals in our series compare favorably to AIS and ASIA motor improvement in historical controls [11-16]. A randomized controlled trial of <24 hours versus 24-72 hours decompression by Haghnegahdar et al. found that 45.9% of individuals undergoing early decompression improved one AIS grade [13]. A 2018 study by Wilson et al. showed that early surgery <24 hours for thoracolumbar SCI resulted in 7 points greater recovery in ASIA lower extremity motor scores in comparison to later surgery, which was statistically significant [16].

One concern with ultra-early or early decompression for SCI is the increased surgical risk or morbidity/mortality with the expedited surgery. There were no surgical complications in our group with ultra-early decompression or postoperative complications directly attributable to ultra-early surgery. A recent study by Guttman et al. revealed that late decompression was associated with higher complications than decompression <24 hours [17]. Our data supports the notion that even earlier decompression, <5 hours, can also be safe without increased risk for complications. The overall postoperative complication rate is higher in this sample than the 24.2% quoted in the STASCIS trial for their early decompression group [5]. However, many of the complications in our sample were medical complications not related to surgery, and the small sample size may also falsely elevate the overall complication rate.

While earlier targets for decompression following SCI are actively being studied, the literature evaluating ultra-early decompression is still sparse, particularly for thoracolumbar SCI. A study by Mattiassich et al. compared <5-hour decompression to 5-24-hour decompression in their cohort of cervical SCI from the Austrian Spinal Cord Injury Study Database [8]. They impressively operated on 33 individuals within 5 hours. They note that the Austrian healthcare system provides the ability for quick trauma surgery. Such early surgical benchmarks may have more barriers in different healthcare systems. They found no significant benefit with ultra-early versus early decompression, and in fact, had a statistically better improvement in AIS grade improvement at follow-up with the early group.

For ultra-early decompression following thoracolumbar SCI, two recent studies out of Germany have investigated decompression within 4 hours in their single-institution cohort of traumatic SCI [18,19]. A 2016 study by Biglari et al. included 51 individuals operated on within 24 hours, of whom 29 were operated on within 4 hours [18]. About 58.6% of their ultra-early sample were thoracolumbar injuries. There was a trend towards improvement in neurologic status as determined by AIS grade, with 44.8% improvement in the <4-hour group compared to 36.4% in the 4-24-hour group, but this was not significant (p=0.4). This study, while including thoracolumbar individuals, did not analyze improvement in the subgroup of thoracolumbar SCI. The same group in Germany published a more recent study with a larger sample size, again comparing ultra-early to early decompression [19]. Their sample of 69 subjects had 46 undergoing ultra-early surgery. Thoracolumbar levels accounted for 60.1% of their ultra-early sample. Again, they found a trend towards improvement in ultra-early decompression for the ultra-early group with 34.8% experiencing improvement in AIS grade compared to 21.7% in the early group, but this did not reach significance (p=0.11). In this study, Bock et al. did perform a subgroup analysis of different injury levels [19]. They found lumbar injuries were most likely to improve neurologically, while thoracic level was least likely to improve. However, they did not analyze improvement in ultra-early versus early decompression by injury level.

Prior ultra-early SCI decompression studies thus only have limited data for thoracolumbar SCI. Another substantial difference in our current series from the published literature is that our sample comes from a level 1 trauma center in the United States. The healthcare system and trauma care in the United States differ greatly from healthcare in Austria or Germany, where the above studies were based. The authors of the prior ultra-early SCI studies note that their hospitals and health systems aided their ability to perform ultra-early decompression. Our case series thus shows the feasibility and safety of ultra-early decompression of thoracolumbar SCI in the United States.

Limitations

This is a small retrospective case series and is subject to many limitations as such. It is hard to draw significant conclusions based on just four ultra-early cases. However, there is still great value in this uncontrolled case series as a “proof-of-concept” that ultra-early decompression in thoracolumbar SCI is feasible [20]. Our case series highlights a rare/understudied treatment of ultra-early decompression, and thus adds a foundation that can be built upon with future literature.

This ultra-early sample included only thoracolumbar injuries, and thus we are unable to extrapolate regarding cervical SCI. Follow-up times also varied greatly, with one individual lost to follow-up after one month. It is possible that a longer follow-up time would reveal additional changes or improvements in neurological function, or delayed complications from surgery. Larger case studies and meta-analyses are needed to better evaluate the superiority of ultra-early versus early decompression in thoracolumbar SCI.

The location of our institution in a major metropolitan area within the United States also limits generalizability. Health system organizations and spine trauma availability/capability in other countries may widely vary.

## Conclusions

Earlier time to decompression in SCI has been linked to improved neurological outcomes in both cervical and thoracolumbar SCI. This case series supports ultra-early decompression as soon as safely possible (within 5 hours from injury) as a target for thoracolumbar SCI which may improve outcomes without added morbidity. However, there exist many logistical barriers to ultra-early surgery. Future studies with larger samples should build upon this data to define further the ideal target time for surgical decompression in thoracolumbar SCI.
